# Supplementary material for: The landscape configuration of zoonotic transmission of Ebola virus disease in West and Central Africa: interaction between population density and vegetation cover
Source: PeerJ. 2015 Jan 20;3:e735. doi: 10.7717/peerj.735 (PMC4304850; doi:10.7717/peerj.735)
Supplement: Table S1 [file peerj-03-735-s001.docx]

Supplemental Table 1. Ebola virus disease outbreaks

| Outbreak Year | Location^1^ | Virus species^2^ | Source |
| --- | --- | --- | --- |
| June-November 1976 | Nzara, South Sudan | SUDV | (WHO, 1978a) |
| September-October 1976 | Yambuku, DRC | EBOV | (WHO, 1978b) |
| June 1977 | Bonduni, DRC | EBOV | (Heymann et al., 1980) |
| July-October 1979 | Nzara, South Sudan | SUDV | (Baron, McCormick, & Zubeir, 1983) |
| November 1994 | Tai Forest, Côte d’Ivoire | TAFV | (Formenty et al., 1999; Le Guenno et al., 1995) |
| November 1994-February 1995 | Mekouka, Gabon | EBOV | (Amblard et al., 1997; Georges et al., 1999; Milleliri, Tévi-Benissan, Baize, Leroy, & Georges-Courbot, 2004) |
|  | Andock, Gabon |  |  |
| January-July 1995 | Kikwit (Mwembe Forest), DRC | EBOV | (Khan et al., 1995; Muyembe & Kipasa, 1995) |
| January-March 1996 | Mayibout 2, Gabon | EBOV | (Georges et al., 1999; Milleliri et al., 2004) |
| July 1996-January 1997 | Booue, Gabon | EBOV | (Georges et al., 1999; Milleliri et al., 2004) |
| October 2000-February 2001 | Rwot-Obillo, Uganda | SUDV | (Lamunu et al., 2004; Okware et al., 2002; WHO, 2001) |
| October 2001-March 2002 | Memdemba, Gabon | EBOV | (Milleliri et al., 2004; D Nkoghe et al., 2005; Pourrut et al., 2005; WHO, 2003a) |
|  | Abolo, ROC |  |  |
|  | Ambomi, ROC |  |  |
|  | Ekata, Gabon |  |  |
|  | Oloba, Congo |  |  |
|  | Etakangaye, Gabon |  |  |
|  | Grand Etoumbi, Gabon |  |  |
| December 2002-April 2003 | Yembelangoye, ROC | EBOV | (Pourrut et al., 2005; WHO, 2003b) |
|  | Mvoula, ROC |  |  |
| October-December 2003 | Mbandza, ROC | EBOV | (Boumandouki et al., 2005) |
| April-June 2004 | Yambio(Forest), South Sudan | SUDV | (Onyango et al., 2007; WHO, 2005) |
| April-May 2005 | Odzala National Park, ROC | EBOV | (Dieudonné Nkoghe, Kone, Yada, & Leroy, 2011) |
| May-November 2007 | Mombo Mounene 2, DRC | EBOV | (Leroy et al., 2009) |
| August-December 2007 | Kabango, Uganda | BDBV | (MacNeil et al., 2010; Towner et al., 2008; Wamala et al., 2010) |
| November 2008-February 2009 | Luebo, DRC | EBOV | (Grard et al., 2011) |
| May 2011 | Nakisamata, Uganda | SUDV | (Shoemaker et al., 2012) |
| July-November 2012 | Isiro, DRC | BDBV | (WHO, 2012a) |
| July-October 2012 | Nyanswiga, Uganda | SUDV | (WHO, 2012b) |
| November2012-January 2013 | Luwero District, Uganda | SUDV | (WHO, 2012c) |
| December 2013 to present | Meliandou, Guinea | EBOV | (Baize et al., 2014; Bausch & Schwarz, 2014) |
| August 2014 | Inkanamongo, DRC | EBOV | (Maganga et al., 2014) |

1 DRC: Democratic Republic of the Congo; ROC: Republic of Congo. 2 SUDV: *Sudan ebolavirus*; EBOV: *Zaire ebolavirus*; TAFV: *Tai Forest ebolavirus*; BDBV: *Bundibugyo ebolavirus*

References

Amblard, J., Obiang, P., Edzang, S., Prehaud, C., Bouloy, M., & Guenno, B. L. (1997). Identification of the Ebola virus in Gabon in 1994. *Lancet*, *349*(9046), 181–2. doi:10.1016/S0140-6736(05)60984-1

Baize, S., Pannetier, D., Oestereich, L., Rieger, T., Koivogui, L., Magassouba, N., … Günther, S. (2014). Emergence of Zaire Ebola Virus Disease in Guinea - Preliminary Report. *The New England journal of medicine*, 1–8. doi:10.1056/NEJMoa1404505

Baron, R. C., McCormick, J. B., & Zubeir, O. A. (1983). Ebola virus disease in southern Sudan: hospital dissemination and intrafamilial spread. *Bulletin of the World Health Organization*, *61*(6), 997–1003. Retrieved from http://www.pubmedcentral.nih.gov/articlerender.fcgi?artid=2536233&tool=pmcentrez&rendertype=abstract

Bausch, D. G., & Schwarz, L. (2014). Outbreak of Ebola Virus Disease in Guinea: Where Ecology Meets Economy. *PLoS Neglected Tropical Diseases*, *8*(7), e3056. doi:10.1371/journal.pntd.0003056

Boumandouki, P., Formenty, P., Epelboin, A., Campbell, P., Atsangandoko, C., Allarangar, Y., … Feldmann, H. (2005). [Clinical management of patients and deceased during the Ebola outbreak from October to December 2003 in Republic of Congo]. *Bulletin de la Société de pathologie exotique (1990)*, *98*(3), 218–23. Retrieved from http://www.ncbi.nlm.nih.gov/pubmed/16267964

Formenty, P., Boesch, C., Wyers, M., Steiner, C., Donati, F., Dind, F., … Le Guenno, B. (1999). Ebola virus outbreak among wild chimpanzees living in a rain forest of Côte d’Ivoire. *The Journal of infectious diseases*, *179 Suppl* , S120–6. doi:10.1086/514296

Georges, A. J., Leroy, E. M., Renaut, A. A., Benissan, C. T., Nabias, R. J., Ngoc, M. T., … Georges-Courbot, M. C. (1999). Ebola hemorrhagic fever outbreaks in Gabon, 1994-1997: epidemiologic and health control issues. *The Journal of infectious diseases*, *179 Suppl* , S65–75. doi:10.1086/514290

Grard, G., Biek, R., Tamfum, J.-J. M., Fair, J., Wolfe, N., Formenty, P., … Leroy, E. (2011). Emergence of divergent Zaire ebola virus strains in Democratic Republic of the Congo in 2007 and 2008. *The Journal of infectious diseases*, *204 Suppl* (Suppl 3), S776–84. doi:10.1093/infdis/jir364

Heymann, D. L., Weisfeld, J. S., Webb, P. A., Johnson, K. M., Cairns, T., & Berquist, H. (1980). Ebola hemorrhagic fever: Tandala, Zaire, 1977-1978. *The Journal of infectious diseases*, *142*(3), 372–6. Retrieved from http://www.ncbi.nlm.nih.gov/pubmed/7441008

Khan, A. S., Tshioko, F. K., Heymann, D. L., Guenno, B. Le, Nabeth, P., Kerstie, B., … Lutte, C. De. (1995). The Reemergence of Ebola Hemorrhagic Fever , Democratic Republic of the Congo , 1995, 76–86.

Lamunu, M., Lutwama, J. ., Kamugisha, J., Opio, a, Nambooze, J., Ndayimirije, N., & Okware, S. (2004). Containing a haemorrhagic fever epidemic: the Ebola experience in Uganda (October 2000–January 2001). *International Journal of Infectious Diseases*, *8*(1), 27–37. doi:10.1016/j.ijid.2003.04.001

Le Guenno, B., Formenty, P., Formentry, P., Wyers, M., Gounon, P., Walker, F., & Boesch, C. (1995). Isolation and partial characterisation of a new strain of Ebola virus. *Lancet*, *345*(8960), 1271–4. Retrieved from http://www.ncbi.nlm.nih.gov/pubmed/7746057

Leroy, E. M., Epelboin, A., Mondonge, V., Pourrut, X., Gonzalez, J.-P., Muyembe-Tamfum, J.-J., & Formenty, P. (2009). Human Ebola outbreak resulting from direct exposure to fruit bats in Luebo, Democratic Republic of Congo, 2007. *Vector borne and zoonotic diseases (Larchmont, N.Y.)*, *9*(6), 723–8. doi:10.1089/vbz.2008.0167

MacNeil, A., Farnon, E. C., Wamala, J., Okware, S., Cannon, D. L., Reed, Z., … Rollin, P. E. (2010). Proportion of deaths and clinical features in Bundibugyo Ebola virus infection, Uganda. *Emerging infectious diseases*, *16*(12), 1969–72. doi:10.3201/eid1612.100627

Maganga, G. D., Kapetshi, J., Berthet, N., Ilunga, B. K., M D, F. K., Kingebeni, P. M., … Leroy, E. M. (2014). Ebola Virus Disease in the Democratic Republic of Congo. *The New England journal of medicine*. doi:10.1056/NEJMoa1411099

Milleliri, J. M., Tévi-Benissan, C., Baize, S., Leroy, E., & Georges-Courbot, M. C. (2004). [Epidemics of Ebola haemorrhagic fever in Gabon (1994-2002). Epidemiologic aspects and considerations on control measures]. *Bulletin de la Société de pathologie exotique (1990)*, *97*(3), 199–205. Retrieved from http://www.ncbi.nlm.nih.gov/pubmed/15462203

Muyembe, T., & Kipasa, M. (1995). Ebola haemorrhagic fever in Kikwit, Zaire. International Scientific and Technical Committee and WHO Collaborating Centre for Haemorrhagic Fevers. *Lancet*, *345*(8962), 1448. Retrieved from http://www.ncbi.nlm.nih.gov/pubmed/7760645

Nkoghe, D, Formenty, P., Leroy, E. M., Nnegue, S., Edou, S. Y. O., Ba, J. I., … Mve, M. T. (2005). [Multiple Ebola virus haemorrhagic fever outbreaks in Gabon, from October 2001 to April 2002]. *Bulletin de la Société de pathologie exotique (1990)*, *98*(3), 224–9. Retrieved from http://www.ncbi.nlm.nih.gov/pubmed/16267965

Nkoghe, Dieudonné, Kone, M. L., Yada, A., & Leroy, E. (2011). A limited outbreak of Ebola haemorrhagic fever in Etoumbi, Republic of Congo, 2005. *Transactions of the Royal Society of Tropical Medicine and Hygiene*, *105*(8), 466–72. doi:10.1016/j.trstmh.2011.04.011

Okware, S. I., Omaswa, F. G., Zaramba, S., Opio, A., Lutwama, J. J., Kamugisha, J., & Rwaguma, E. B. (2002). An outbreak of Ebola in Uganda, *7*(12), 1068–1075.

Onyango, C. O., Opoka, M. L., Ksiazek, T. G., Formenty, P., Ahmed, A., Tukei, P. M., … Rollin, P. E. (2007). Laboratory diagnosis of Ebola hemorrhagic fever during an outbreak in Yambio, Sudan, 2004. *The Journal of infectious diseases*, *196 Suppl* , S193–8. doi:10.1086/520609

Pourrut, X., Kumulungui, B., Wittmann, T., Moussavou, G., Délicat, A., Yaba, P., … Leroy, E. M. (2005). The natural history of Ebola virus in Africa. *Microbes and infection / Institut Pasteur*, *7*(7-8), 1005–14. doi:10.1016/j.micinf.2005.04.006

Shoemaker, T., MacNeil, A., Balinandi, S., Campbell, S., Wamala, J. F., McMullan, L. K., … Nichol, S. T. (2012). Reemerging Sudan Ebola virus disease in Uganda, 2011. *Emerging infectious diseases*, *18*(9), 1480–3. doi:10.3201/eid1809.111536

Towner, J. S., Sealy, T. K., Khristova, M. L., Albariño, C. G., Conlan, S., Reeder, S. a, … Nichol, S. T. (2008). Newly discovered ebola virus associated with hemorrhagic fever outbreak in Uganda. *PLoS pathogens*, *4*(11), e1000212. doi:10.1371/journal.ppat.1000212

Wamala, J. F., Lukwago, L., Malimbo, M., Nguku, P., Yoti, Z., Musenero, M., … Okware, S. I. (2010). Ebola hemorrhagic fever associated with novel virus strain, Uganda, 2007-2008. *Emerging infectious diseases*, *16*(7), 1087–92. doi:10.3201/eid1607.091525

WHO. (1978a). Ebola haemorrhagic fever in Sudan, 1976. Report of a WHO/International Study Team. *Bulletin of the World Health Organization*, *56*(2), 247–70. Retrieved from http://www.pubmedcentral.nih.gov/articlerender.fcgi?artid=2395561&tool=pmcentrez&rendertype=abstract

WHO. (1978b). Ebola haemorrhagic fever in Zaire, 1976. *Bulletin of the World Health Organization*, *56*(2), 271–93. Retrieved from http://www.pubmedcentral.nih.gov/articlerender.fcgi?artid=2395567&tool=pmcentrez&rendertype=abstract

WHO. (2001). Outbreak of Ebola haemorrhagic fever, Uganda, August 2000-January 2001. *Relevé épidémiologique hebdomadaire / Section d’hygiène du Secrétariat de la Société des Nations = Weekly epidemiological record / Health Section of the Secretariat of the League of Nations*, *76*(6), 41–6. Retrieved from http://www.ncbi.nlm.nih.gov/pubmed/11233580

WHO. (2003a). Outbreak(s) of Ebola haemorrhagic fever, Congo and Gabon, October 2001-July 2002. *Relevé épidémiologique hebdomadaire / Section d’hygiène du Secrétariat de la Société des Nations = Weekly epidemiological record / Health Section of the Secretariat of the League of Nations*, *78*(26), 223–8. Retrieved from http://www.ncbi.nlm.nih.gov/pubmed/15571171

WHO. (2003b). Outbreak(s) of Ebola haemorrhagic fever in the Republic of the Congo, January-April 2003. *Relevé épidémiologique hebdomadaire / Section d’hygiène du Secrétariat de la Société des Nations = Weekly epidemiological record / Health Section of the Secretariat of the League of Nations*, *78*(33), 285–9. Retrieved from http://www.ncbi.nlm.nih.gov/pubmed/14509121

WHO. (2005). Outbreak of Ebola haemorrhagic fever in Yambio, south Sudan, April - June 2004. *Relevé épidémiologique hebdomadaire / Section d’hygiène du Secrétariat de la Société des Nations = Weekly epidemiological record / Health Section of the Secretariat of the League of Nations*, *80*(43), 370–5. Retrieved from http://www.ncbi.nlm.nih.gov/pubmed/16285261

WHO. (2012a). WHO | Ebola outbreak in Democratic Republic of Congo. Retrieved from http://www.who.int/csr/don/2012_08_18/en/

WHO. (2012b). WHO | Ebola in Uganda. Retrieved from http://www.who.int/csr/don/2012_07_29/en/

WHO. (2012c). WHO | Ebola in Uganda 2. Retrieved from http://www.who.int/csr/don/2012_11_17/en/
